# Using Text Messaging to Improve Access to Prenatal Health Information in Urban African American and Afro-Caribbean Immigrant Pregnant Women: Mixed Methods Analysis of Text4baby Usage

**DOI:** 10.2196/14737

**Published:** 2020-02-13

**Authors:** Tenya M Blackwell, LeConte J Dill, Lori A Hoepner, Laura A Geer

**Affiliations:** 1 Department of Environmental and Occupational Health Sciences SUNY Downstate Health Sciences University School of Public Health Brooklyn, NY United States; 2 Department of Social and Behavioral Sciences College of Global Public Health New York University New York, NY United States

**Keywords:** Text4baby, mHealth, pregnancy, text messaging, health information, prenatal health, disparities

## Abstract

**Background:**

The Text4baby (T4B) mobile health (mHealth) program is acclaimed to provide pregnant women with greater access to prenatal health care, resources, and information. However, little is known about whether urban African American and Afro-Caribbean immigrant pregnant women in the United States are receptive users of innovative health communication methods or of the cultural and systematic barriers that inhibit their behavioral intent to use T4B.

**Objective:**

This study aimed to understand the lived experiences of urban African American and Afro-Caribbean immigrant pregnant women with accessing quality prenatal health care and health information; to assess usage of mHealth for seeking prenatal health information; and to measure changes in participants’ knowledge, perceptions, and behavioral intent to use the T4B mHealth educational intervention.

**Methods:**

An exploratory sequential mixed methods study was conducted among pregnant women and clinical professionals for a phenomenological exploration with focus groups, key informants, interviews, and observations. Qualitative themes were aligned with behavioral and information technology communications theoretical constructs to develop a survey instrument used. repeated-measures pre- and post-test design to evaluate changes in participants’ knowledge, attitudes, and beliefs, of mHealth and T4B after a minimum of 4 weeks’ exposure to the text message–based intervention. Triangulation and mixing of both qualitative and quantitative data occurred primarily during the survey development and also during final analysis.

**Results:**

A total of 9 women participated in phase 1, and 49 patients signed up for T4B and completed a 31-item survey at baseline and again during follow-up. Three themes were identified: (1) patient-provider engagement, (2) social support, and (3) acculturation. With time as a barrier to quality care, inadequate patient-provider engagement left participants feeling indifferent about the prenatal care and information they received in the clinical setting. Of 49 survey participants, 63% (31/49) strongly agreed that T4B would provide them with extra support during their pregnancy. On a Likert scale of 1 to 5, participants’ perception of the usefulness of T4B ranked at 4.26, and their perception of the compatibility and relative advantage of using T4B ranked at 4.41 and 4.15, respectively. At follow-up, there was a 14% increase in participants reporting their intent to use T4B and a 28% increase from pretest and posttest in pregnant women strongly agreeing to speak more with their doctor about the information learned through T4B.

**Conclusions:**

Urban African American and Afro-Caribbean immigrant pregnant women in Brooklyn endure a number of social and ecological determinants like low health literacy, income, and language that serve as barriers to accessing quality prenatal health care and information, which negatively impacts prenatal health behaviors and outcomes. Our study indicates a number of systematic, political, and other microsystem-level factors that perpetuate health inequities in our study population.

## Introduction

### Poor Birth Outcomes in Brooklyn, New York

Women and children of color in Brooklyn, New York, suffer inequities in health because of disproportionately higher rates of adverse birth outcomes such as low birth weight (LBW) and preterm birth. In 2014, the overall LBW (<2500 kg) rate for Brooklyn was 8.2% compared with 8.5% for all of New York City and 8.1% for the state of New York [1,2]. In 2012, the national rate for LBW was at 7.99% [3]. African American women have a 3 to 4 times higher risk than non-Hispanic/Latino whites for adverse infant health outcomes such as LBW [4], and according to Martins et al, infants born to non-Hispanic black women have the highest rates of LBW (13.1%), 2 or more times greater than that for infants born to women of other race and ethnic groups [4].

### The Role of Communication

Health communication researchers attest that the public health community has a limited understanding of what health communication can offer to the elimination of health inequities [5]. Evidence shows that health communication can increase the intended audience’s knowledge and awareness of a health issue, problem, or solution; influence perceptions, beliefs, and attitudes that may change social norms; prompt action; demonstrate or illustrate healthy skills; reinforce knowledge, attitudes, or behavior; show the benefit of behavior change; advocate a position on a health issue or policy; increase demand or support for health services; refute myths and misconceptions; and strengthen organizational relationships [5].

However, Freimuth and Quinn assert that health communication alone, without environmental support, is not effective at sustaining behavioral changes at the individual level [6]. High-quality communication and a positive patient-provider relationship are critical components of patient-centered quality care [7]. Furthermore, engaged patients who communicate with their providers are more likely to be treated with respect, receive adequate health information, and engage in health behaviors such as physical activity and healthy dietary behaviors [8,9].

### Pregnant Women and Mobile Health

Mobile health (mHealth) has evolved as the branch of electronic health broadly defined as the use of mobile computing and communication technologies in health care and public health [10]. It has over the last decade become a new tool used in the delivery of health services for disease management and prevention in a variety of health arenas and as an innovative means to supplement traditional health communications targeting doctors, nurses, patients, or even the lay population [11].

Available literature displays use of mHealth for smoking cessation [12], physical activity [13], diet and weight loss [14], and managing chronic disease such as diabetes [15]. mHealth text messaging services (SMS) have impacted pregnant women in a number of ways.

Research in Canada, Saudi Arabia, and Argentina show pregnant women positively benefiting from the use of mHealth through increased access to prenatal health services, improved information-seeking behaviors, and has provided support throughout pregnancy with increased prenatal health knowledge and improved access to care [16-18]. Pregnant women or those caring for their first child are highly likely to use mHealth to increase their prenatal health information–seeking behaviors as they have a stronger need and desire to obtain pregnancy- and child health–related information [8,19,20].

Much of the current literature around mHealth for pregnant women examines participants’ interests, acceptance, and the feasibility of text messaging for improving perinatal and postnatal care. For many immigrant populations, language and speech are important factors of consideration for any health communication endeavor either through providers or through technology. A recent cross-sectional study in Germany highlights the importance of culturally tailored text messaging and the consideration of users’ health beliefs and health literacy levels in message development [21]. Similarly, in a systematic review, researchers underline the importance of the accommodation of local languages and preferences in the content of effective text messaging programs [22].

Dobson et al’s qualitative study corroborates the benefits of culturally tailored mHealth programs for improved diet and exercise in pregnant women [22]; the feasibility and acceptability of a text messaging program aimed at smoking cessation for pregnant women [16] demonstrates that high acceptance and perceived feasibility of mHealth indicate a willingness to use and benefit from such services.

These studies provide a framework for this work and depict the need to first understand users’ perceptions, acceptance, and overall intent to use mHealth for the purpose of accessing prenatal health information.

### Text4baby

Despite a high level of activity and interest around text messaging apps, the documented evidence on their effectiveness remains limited [23]. The Text4baby (T4B) program was designed to offer support, improve health literacy, increase expectations for successful pregnancy, build the knowledge and skills to manage one’s own health, and prevent health risks by avoiding behavioral risk factors including smoking and drinking. Launched in 2012, it is a US mHealth information text messaging service led by the US Centers for Disease Control and Prevention that sends free text messages to women who are pregnant or have children younger than 1 year, providing them with information and reminders to improve their health and the health of their babies [17].

Research on T4B has focused primarily on the content and frequency of the T4B messages in comparison with messages from other pregnancy-related apps [17]. Enrollment and health literacy among potential T4B participants have also been a focus of T4B evaluation [24], along with its use to promote influenza vaccination among pregnant women [18], and for the design of interventions to improve physical activity in pregnant women [25].

Evans et al [26] emerged as a seminal empirical investigator of the impact of T4B on knowledge and behavioral outcomes of pregnant women. The earliest research published was a pilot study conducted with pregnant women in Fairfax County, Virginia, who presented for care at their local health department [17,27]. Through a randomized controlled trial (RCT), Evans et al found increased odds of participants feeling prepared for motherhood in those exposed to T4B versus normal prenatal care [26,28]. In other works, Evans et al [26] conducted an RCT of a group of military health service participants. Researchers sought to evaluate differences in adequate use of prenatal care, as defined by the Adequacy of Prenatal Care Utilization Index, in T4B participants compared with participants not receiving the T4B messages; however, others attest the study’s ability to accurately measure true behavior change [27].

Effective health behavioral change programs should be guided by strong theoretical models [29-32]. To date, few mHealth and text messaging studies have adequately incorporated the use of theory to examine the impact, acceptance, feasibility, and behavioral intent to use mHealth. The current landscape of mHealth and T4B research using information technology (IT) theories is limited [33]. There are many factors that can influence the use of technology as a channel for prenatal health information within low-income urban and immigrant populations, and researchers strongly point to the need for multidisciplinary frameworks that capture the complexities of using mobile sources in health information behaviors [34].

**Figure 1:**
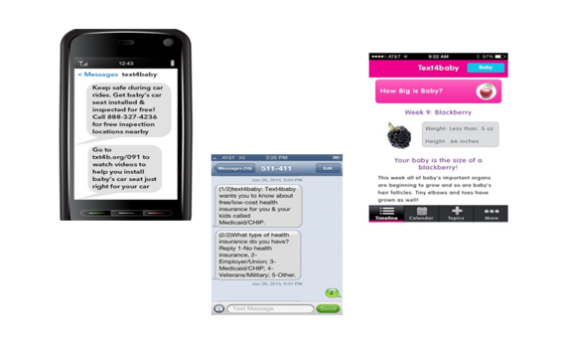
Screenshot of Text4baby messages.

Marton and Chun [35] demonstrate that an integration of theoretical perspectives from the health sciences, social sciences, communication, and information sciences research is necessary to fully understand this complex behavior. This study will leverage theoretically motivated constructs from research in consumer behavior and health information and communications technology to assess participants’ knowledge, attitudes, beliefs, and behavioral intent to use T4B. Our research will add to the current body of literature around T4B by first assessing its impact on participants’ perceptions of its feasibility, acceptance, compatibility, and usefulness. We seek to further fill empirical gaps by utilizing theoretically motivated constructs to examine our study populations’ intent to use the T4B program for prenatal health information. This will allow practitioners and program developers to predict the use of the T4B program in this population to design better strategies that encourage its use for maternal health education and risk communication in ethnically, culturally, and socioeconomically diverse immigrant communities in Brooklyn. Therefore, our research demonstrates how theory and explicit testing of mediators can be used for evaluations of T4B [36]. See Figure 1.

### Theoretical Underpinnings

Previous works on the individual adoption of ITs have identified that a number of consumer characteristics and perceptions influence adoption of IT [13]. A recent systematic review of consumer health technology acceptance research points to studies that have assessed the effects of age, income, and education on health technology acceptance; however, theoretical constructs have not yet been fully considered in consumer health technology acceptance studies [13,37]. A combination of the Theory of Planned Behavior [38,39] and Technology Acceptance Model was used to examine the influence of participants’ subjective norms and perceived behavioral control (attitudes and beliefs) on their ability and intention to use T4B [40,41]. Constructs from Roger’s Diffusion of Innovation Theory were also explored in identifying valuable predictors for T4B intent [42]. In this research, our goal was to understand what it is like to be an urban and/or immigrant pregnant woman with accessing prenatal health care and information in Brooklyn, New York, and to utilize behavioral and technology assimilation of theoretical constructs in tandem with qualitative data to develop a survey instrument to measure pregnant women’s knowledge, perceptions, and behavioral intent to use the T4B health communication program.

The overall purpose of this study was to test a maternal health education intervention (T4B) to see if it improves access to prenatal health care and information, improves prenatal health-seeking behaviors in pregnant women, and determines the likelihood that pregnant women in central Brooklyn would adopt T4B as a viable channel for prenatal health information. The underlying assumptions are that patients’ knowledge about mHealth and T4B, their attitudes toward text messaging for prenatal health, their subjective and normative beliefs about prenatal health information sources, their perceptions on the usefulness and compatibility of T4B, and factors such as satisfaction and visibility of T4B will affect their acceptance of and behavioral intent to use the T4B program for improved access to prenatal and maternal health care and information.

### Research Site

Study participants were recruited from the SUNY Downstate Medical Center University Hospital located in the East Flatbush section of Brooklyn, New York. East Flatbush is a community located in the central region of Brooklyn with a population of 154,575 persons. A total of 88.00% (136,026/154,575) of the population of East Flatbush is black, with 53% of residents born outside the United States, and almost 10% are reported to have limited English proficiency. In East Flatbush, 15.6% of live births receive late or no prenatal care, and according to the NYC Department of Health and Mental Hygiene, 1 in 8 births in this population are delivered preterm. The Maternal Fetal Medical Division of the Department of Obstetrics and Gynecology at Downstate provides perinatal and gynecological services for pregnant and nonpregnant black and Afro-Caribbean women. This location was chosen because of its vastly diverse urban and immigrant black population with migrants from a number of Afro-Caribbean countries including Haiti; Trinidad; and Jamaica, West Indies. Our research at this location offers an opportunity to study different social and cultural perspectives from subgroups within the black community and how these differences shape pregnant women’s experiences in Brooklyn, New York. This site is also a location where scientists, physicians, and researchers hold expertise in risk communication of reproductive health issues, perinatal epidemiology, and environmental exposure assessment specifically with the use of biological markers. Geer et al, while characterizing important environmental risk factors in our target population, have indicated a need for further study and exposure reduction efforts tailored specifically to this community [43]. Our research at this site will expound the knowledge on innovative risk communication and health promotion efforts that are most suitable and receptive for the population of pregnant women. The authors have chosen not to use a pseudonym for the research site/research partner. Some scholars [44] agree that removing identifying information erases important contextual information that is valuable to the research. To not anonymize location of the research recognizes that SUNY Downstate sits within specific social, historical, cultural, environmental, geographical, and symbolic moments and meanings [45].

The study and protocol were approved by the Institutional Review Boards of the State University of New York Downstate Medical Center. Each participant signed an informed consent form before participation.

This study used multiple methods of inquiry including both qualitative phenomenology and IT constructs to explore the views of pregnant women in Brooklyn, New York, on prenatal health care and text messaging programs such as T4B to inform the development of a quantitative instrument to measure changes in their knowledge, attitudes, beliefs, and intent to use T4B.

## Methods

### Overview

A sequential mixed approach [46] was used to first gain knowledge about the experiences of urban African American and Afro-Caribbean immigrant pregnant women with accessing prenatal health care and prenatal health information at an urban metropolitan health center in New York City. We also sought to understand participants’ perceptions about the use of mHealth and the T4B text messaging program as a source of prenatal health information and resources. We then conducted a repeated-measures pre- and post-test design study to measure changes in participants’ knowledge, attitudes, and beliefs on key prenatal health behaviors, perceptions, and intent to use the T4B text messaging program.

#### Recruitment and Sampling

The sampling techniques for the qualitative phase were driven by the study’s socioecological framework, which was used to aid in the exploration and discovery of factors that serve as barriers or facilitators of access to prenatal care and the use of mHealth communications among pregnant women in this community. Sample participants were pregnant women receiving prenatal care and clinical providers of prenatal health care services at SUNY Downstate. Various nonprobability sampling techniques were used during the early phase of inquiry. We used purposeful maximum variation sampling to recruit pregnant participants who (1) were aged 18 to 45 years, (2) owned a cell phone with text messaging capabilities, and (3) could communicate fluently in English. Creswell and Plano Clark [47] render that maximum variation sampling captures the variation in experiences and perspectives from study participants. They further specify that if participants are purposefully chosen to be different at onset, then the variation in views will be reflected and will provide a more comprehensive picture of the phenomena under study [47]. We also chose purposive sample for participants who were able to communicate fluently in English as we found that participants from our target population who were not proficient in speaking or reading English showed difficulty in understanding consent forms and pretest survey questions. Many patients at Downstate who primarily spoke Haitian creole attempted to use a mobile interpretation app to translate the survey but naturally were unsuccessful. Therefore, we only recruited participants with adequate English proficiency. Expert sampling is a type of purposive sampling technique that is used as expert elicitation—acquiring knowledge from professionals who possess a particular expertise [48]. We used this form of purposive sampling to select clinicians from the obstetrics and gynecology clinic at Downstate Medical Center with experience providing prenatal care services to our study population as key informants to our study.

Their expert perspective helped broaden our scope of understanding the experiences of pregnant women through the eyes of both patients and providers.

A total of 22 participants agreed to be in the study; however, 9 women were successfully recruited and participated in 2 focus groups, 1 one-on-one interview, and 2 key informant interviews. For phenomenological research, Creswell et al [49] recommend a range of 5 to 25 participants; Fitzgerald et al [50] recommend a minimum sample size of 6. Our overall sample size of 9 falls within the recommendations of these qualitative research scholars. Moreover, 7 of the 9 participants were patients at the clinic.

The 2 key informants were clinical staff yielding a total of 9 participants for the descriptive phenomenology. Participants were directly approached by the study investigator while waiting to be seen at the clinic. They were initially recruited to participate in semistructured focus groups; however, difficulty with coordinating and scheduling focus groups at the convenience of the pregnant patients led to one-on-one in-depth interviews with patients as an alternative for data collection. Qualitative data collection took place from March 2016 to June 2016.

A standard demographic survey was completed during the consent process to gather data on participant age, education level, country of origin, race, ethnicity, insurance provider, and marital status. A total of 2 discussion guides were created for patients and providers to guide the focus groups and interviews with open-ended questions and probes to introduce selected a priori themes: (1) access and barriers to prenatal health care and information; (2) health disparities and the built environment; (3) cultural, familial, and social relationships; (4) knowledge, attitudes, beliefs, and use of mHealth and T4B; and (5) health information–seeking behaviors and sources.

#### Qualitative Data Collection

Focus groups and one-on-one interviews with pregnant women took place in a secured location at Downstate, and for convenience, they were scheduled to coincide with patients’ prenatal visits. Key informant interviews took place at informant’s offices. Interviews averaged between 60 and 90 min, with time allotted for refreshments for the pregnant participants. Participants gave oral responses to the set of open-ended questions. We completed a total of 2 focus groups and 1 in-depth interview with patients and 2 separate key informant interviews with providers. Data collection ended once saturation was reached and no new information emerged as interviews transpired. Interview data were triangulated with 3 patient observations in the natural setting of the clinic environment. Participant observations offer researchers an opportunity to gain a firsthand encounter with the phenomena under interest rather than relying solely on a secondhand account provided by participants [51]. We conducted 3 patient observations in the clinic waiting areas during the data collection phase. We observed patient engagement, attitudes, temperament, and the receipt of prenatal health education provided from a registered nurse educator from Downstate. Participants’ observation also permitted within-method triangulation and increased validation of the dataset [51]. Care was taken to ensure research ethics, protecting patients’ anonymity, confidentiality, and respecting their wishes were met. Moms received a US $20 Target gift card and a round trip metro transit card (worth US $5.50) as incentive. Participants provided written consent to participate and agreed to be audio recorded during the interviews.

#### Quantitative Data Collection

A convenience sample of 49 pregnant women was recruited during standard visits to undergo the T4B mHealth intervention. Inclusion criteria were the same for phases 1 and 2 to include pregnant women receiving care at SUNY Downstate, aged 18 to 45 years, who owned a cell phone with text messaging capabilities and were able to communicate in English. Participants were recruited while waiting for care in either the clinic triage area and while waiting to see the doctor after triage or waiting to receive a sonogram. Thematic findings generated from the qualitative analysis were aligned with constructs from consumer behavior, communications technology, and diffusion theories to develop a 32-item survey for a repeated-measures test of perceived usefulness, perceived behavioral control, and relative advantage of using T4B.

The instrument was a self-administered questionnaire that leveraged the constructs from other validated instruments [27] while also drawing on the suggested theoretical measures used for research on technology acceptance [39,43], consumer behavior [42], and mobile technology diffusion [33,52]. The 32-item survey is a composite of 8 scales representing 8 dependent variables and was administered as a pre-/post-test to assess changes in participants’ perceptions regarding the statements. Following consent, participants were invited to use their mobile phones to enroll in the T4B program and partake in 2 surveys, 1 on the day of recruitment and a second follow-up survey after a minimum of 4 weeks of receiving the text messages. Recruitment for the quantitative phase took place between October 2016 and March 2017 and continued on a rolling basis until the minimum desired number of participants was reached. Upon receiving consent, we administered the pretest survey and then assisted participants to follow the steps for signing up for T4B. After which, participants provided contact information to be reached after 4 weeks to complete a posttest survey during a subsequent prenatal visit. After a minimum of 4 weeks, participants were contacted to coordinate with their next prenatal visit to complete the follow-up survey. The posttest survey was identical to the initial baseline survey with the addition of 1 item to assess participants’ self-report of actual reading of the text messages. Participants received a US $20 gift card and a roundtrip transit card (worth US $5.50) for their participation.

#### Quantitative Measures and Instrumentation

##### Attitudes Scale

This scale contained a battery of questions to assess participants’ attitudes regarding key prenatal health behaviors such as diets, taking prenatal vitamins, smoking, drinking, and seeking prenatal care and information through mHealth. Participants were asked to rate their agreement with a series of statements on a 5-point Likert scale of 1 to 5 from “strongly disagree” to “strongly agree.” The scale contained 8 items. The minimum possible score for the attitude scale was 8, and the maximum score was 40. A higher score was a reflection of a more strongly positive attitude toward the behavioral statements captured in the items.

##### Beliefs Scale

The beliefs scale contained 2 items that measured participants’ subjective norm—the perceptions of family, peers, and persons of influence—on the use of mHealth and T4B to obtain prenatal health information.

The scale had a minimum score of 2 and a maximum score of 10. Variables specific to beliefs were adapted from previous studies of behavioral factors influencing text messaging intention [42]. Example belief variables included the following: “family and friends who are important to me would welcome using Text4baby for prenatal health information,” and they were measured on a 5-point scale ranging from strongly disagree to strongly agree.

##### Perceived Usefulness Scale

The perceived usefulness construct contained 6 items to assess the degree to which participants perceived T4B to be useful to them. The maximum score possible for the scale was 30. Participants were asked to rate their agreement with statements such as “Info from Text4baby will help me ask more questions to the doctors and nurses at the clinic” and “online sources are useful for searching for prenatal health information.”

##### Perceived Ease of Use Scale (Behavioral Control)

A 7-item scale was used to measure participant’s perceived behavioral control for using mHealth and if they find mHealth easy to engage. With a maximum score of 35, example measures included the following: “it is easy for me to get prenatal health information on my mobile phone” and “I have all the skills and knowledge I need to use the Text4baby program.”

##### Compatibility Scale

The compatibility scale was a 2-item scale that contained questions to assess the degree to which participants utilize mobile technology, particularly text messaging to communicate throughout their daily lives. Measured on a 5-point Likert scale, the compatibility scale asked questions such as “I communicate regularly with friends and family through text messages.”

##### Relative Advantage Scale

We wanted to assess whether participants perceived T4B to be advantageous to them for the purposes of acquiring prenatal health information and resources. The relative advantage scale containing 3 items was also measured on a Likert scale. Example measures included “using Text4baby will allow me to reach healthier prenatal health goals” and “Text4baby messages will be a better source of prenatal health information for me.”

##### Visibility Scale

The lack of awareness or visibility of T4B was a huge concept that was discovered during the qualitative phase of this study. Many of the participants had not heard of T4B despite its widespread promotion and local advertisement. We chose to assess visibility with a 2-item scale that contained a battery of questions to assess participants’ agreement on whether they have seen or heard of others using T4B or if people they know depend more on the internet and mHealth for health information.

#### Intent

Unlike other studies [33], we did not assess the strength of the previously mentioned constructs in predicting participants’ behavioral intent to use T4B; however, we measured behavioral intent using 2 items to determine the level of agreement with statements such as “I plan to use Text4baby for prenatal health care and information measured on a 5-point Likert scale of ‘strongly disagree’ to ‘strongly agree’.”

See Tables 1 and 2 for a description of survey questionnaire components and corresponding alpha coefficients.

**Table 1:** Questionnaire components by scale.

Scale	Item	Components measured	Theoretical origin	Response options
Attitude	1-8	Feelings on health behaviors like smoking, drinking, diet, health care utilization	TPB^a^	Strongly disagree–strongly agree
Perceived Usefulness	11-16	Intrinsic motivations to use T4B^b^ due to perceived benefits of using	TAM^c^	Strongly disagree–strongly agree
Perceived Ease of Use	17-23	Behavioral control and abilities to use text messaging for prenatal health info	TPB, TAM	Strongly disagree–strongly agree
Compatibility	24-25	Perceptions whether text messaging and T4B fits into the everyday lives	DOI^d^	Strongly disagree–strongly agree
Relative advantage	26-28	Perceptions of the benefits of using T4B	DOI	Strongly disagree–strongly agree
Visibility	29-30	knowledge and awareness of T4B	DOI	Strongly disagree–strongly agree
Intent	31-32	Plans and intentions to use T4B	TPB, TAM DOI	Strongly disagree–strongly agree

^a^TPB: Theory of Planned Behavior.

^b^T4B: Text4baby.

^c^TAM: Technology Acceptance Model.

^d^DOI: Diffusion of Innovation Theory.

**Table 2:** Cronbach alpha coefficients for questionnaire by scale.

Scale	Alpha coefficient	Items, n	Mean scale rank^a^
Attitude scale	.661	8	—^b^
Beliefs scale	.883	2	4.08
Perceived Usefulness scale	.835	6	4.26
Perceived Ease of Use scale	.718	7	3.95
Compatibility scale	.806	2	4.41
Relative advantage scale	.880	3	4.15
Visibility scale	.193	2	—
Intent scale	.914	2	4.28

^a^Mean rank on a scale of 1-5 strongly disagree–strongly agree analyzed by Wilcoxon sign rank test.

^b^Not applicable.

### Analysis

#### Qualitative Data Analysis

A total of 5 qualitative data sources were generated from the focus groups and interviews. Audio recordings from each interview were transcribed and uploaded using the NVivo (version 11.0 QSR International) [53] qualitative data management software. To ensure analytic rigor, we followed Colaizzi’s 7-step phenomenological approach for extracting, organizing, and analyzing our narrative dataset [54]. With this approach, significant statements made by interviewees were taken from the transcripts and grouped together to formulate themes that describe key elements of experiencing the phenomenon, or area being studied Creswell et al [49]. Significant statements are those most outstanding comments, sentences, or quotes taken from participants that describe how they experienced the phenomenon [54]. Subsequently, similar significant statements are placed into clusters of meanings (or themes).

A total of 392 significant statements were extracted from 5 transcripts and broken into 9 a priori theme clusters. These clusters of significant statements were then coded using coding methods described by Miles et al [55] and analyzed to identify emergent themes.

#### Quantitative Data Analysis

SPSS version 24 (IBM) was used to analyze the quantitative dataset. The 32- items in the instrument were analyzed both as single Likert-type items in which frequency distributions, measures of central tendency, and variance were among the descriptive statistics used to summarize the variables. The 7 subscales were also analyzed as composite Likert scales in which nonparametric tests of comparison were run. Reliability of each scale—defined as how well a set of items within a scale measured the same underlying constructs—was determined based on the internal reliability using Cronbach alpha coefficient [56]. Changes in participants’ attitudes and perceptions as a result of exposure to T4B messages between baseline and follow-up were analyzed using a matched-pairs Wilcoxon sign-ranked test. We chose this statistical test over a paired sample *t* test because of the ordinal nature of the Likert-type subscales.

## Results

### Overview

A total of 58 participants were successfully recruited from the OB/GYN clinic at SUNY Downstate Medical Center for this study. Moreover, 9 participants, including 7 pregnant women and 2 clinicians, participated in the qualitative phase, and 49 pregnant women participated in phase 2 and completed the pretest and posttest surveys. The average age of the participants (n=49) was 28 years. Approximately two-thirds (63%) of the participants were US born, whereas the remaining were born in either Trinidad and Tobago; Haiti; or Jamaica, West Indies (36.7%). In addition, 15 participants (30.6%) reported that they were married, and 65.3% of the participants reported not being married or living with partner. Of the 38 participants, 38.8% had a high school diploma or the equivalent general education diploma, 20.4% attended technical school, and 14.3% reported having a 4-year college degree. A high proportion (87.8%) of participants had public health insurance such as Medicaid or Family Health Plus, whereas 4.1% (n=2) of the participants reported having private insurance through an employer. See Tables 3 and 4 for demographic characteristics of focus group and survey participants.

**Table 3:** Demographic characteristics of patients participating in focus groups and interviews.

Demographics		Value, n (%)
**Maternal age**		
	20-29	4 (57)
	30-39	2 (29)
	40-45	1 (14)
**Maternal education**		
	High school diploma or GED^a^	3 (43)
	Technical school	1 (14)
	College, 4-year degree	3 (43)
**Maternal ethnicity**		
	African American	6 (86)
	Hispanic	1 (14)
**Maternal country of birth**		
	United States	2 (29)
	Jamaica	2 (29)
	Haiti	2 (29)
	Trinidad	1 (13)
**Maternal marital status**		
	Married	0 (0)
	Not married	7 (100)
**Maternal insurance type**		
	Public	6 (86)
	Private	1 (14)

^a^GED: general education diploma.

**Table 4:** Demographic characteristics of patients participating in the survey.

Demographics		Value, n (%)
**Age (years; n=49)**		
	<20	3 (6)
	20-34	38 (77)
	35+	6 (12)
**Education (n=38)**		
	Some high school	2 (4)
	High school diploma or GED^a^	19 (39)
	Technical school	10 (20)
	College, 4-year degree	7 (14)
**Ethnicity (n=47)**		
	African American	22 (45)
	Caribbean West Indian	24 (49)
	Other	1 (2)
**US born (n=49)**		
	Yes	31 (63)
	No	18 (37)
**Marital status (n=47)**		
	Married	15 (31)
	Single	32 (65)
**Insurance (n=45)**		
	Public	43 (88)
	Private	2 (4)

^a^GED: general education diploma.

### Prenatal Experiences With the US Health Care System

A total of 3 major themes were garnered from the interviews and observations: (1) inadequate patient-provider engagement, (2) social support, and (3) acculturation. Our qualitative findings showed that time served as a huge barrier impeding an adequate level of engagement and communication between pregnant women and clinicians at the Downstate prenatal health clinic. Participants reported expending a large amount of time—sometimes more than 4 and 5 hours from arrival to departure waiting for prenatal care. This often left many of them feeling frustrated, impatient, and with a poor temperament regarding the care they receive. One participant described dissatisfaction with her experiences, with waiting times for care creating great amount of frustration with the prenatal health system:

I get here earlier and then you’re still here until 1 in the afternoon you know…Like…I don’t understand that part…

Another participant chimed:

and after…being somewhere for 4 or 5 hours you just wanna eat and go home

This caused huge barriers in communication and engagement between pregnant women and providers. During an observation in the waiting areas, we noticed high levels of frustration marked by signs of huffing and puffing, constant complaints, restlessness, and high irritability, as captured in this observation field note:

Patients were very irate with the wait time – says “its miserable in here.” They report that the doctors are very good and very thorough with providing information and addressing concerns when asked but having to wait so long; being pregnant, tired and hungry made them very angry.

Participant 2 from focus group #2 described the actual amount of time spent in the office with doctors as “like an assembly line”:

I feel, every time I come here I’m drained...I’m there...say the appointment starts from 10 o’clock...I’m there at 8 o’clock...and I’m still there to 1 o’clock...hmp...just to see him for four minutes.

As patients are moved in and out so quickly, women felt as though they are not given enough opportunity to speak with their doctors and ask questions or given sufficient time to engage with providers in a manner that leads to acquiring information or addressing any concerns they may have. They are reluctant to engage. When asked about the relationship between themselves and the doctor, the women in this study perceived that “here…there’s so much of a rush…they don’t put too much time in to do that.” Some participants expressed a desire for clinicians to “be more communicative” and articulated dissatisfaction with their care as captured in the following statement:

Well I think the doctors need to be more…umm like communicative with the patients, not just come and then just check you and then {oh ok everything is fine I will give you like another appointment like next week}…that’s not good.

We found differences of perception between participants who were either US born, who had migrated to the United States less than 2 years, or within 5 years or greater. Discontent over the quality of prenatal care and information received came predominantly from younger participants, those born in the United States, and those more acculturated. Notably, the attitudes and experiences of participants who were newer immigrants were much more positive. Potentially, their increased exposure to the systematic and structural racism known to perpetuate the US health care industry have led to such negative perspective of their prenatal health experiences. With regard to the prenatal care she receives, 1 participant who migrated more recently expressed:

I’m from the Caribbean so…that seems like…top of the class to me…I’m from Trinidad…so I am content, it would too that I have never seen better than this. So my experience would be different so to me its ok…its great

During the key informant interviews, clinical providers described the practices at Downstate and reported that immigrant and pregnant women have a great deal of access to prenatal care through various insurance programs such as New York State Medicaid and other pregnancy assistance programs such as Prenatal Care Assistance Program (PCAP), a prenatal care program developed to provide comprehensive perinatal care to low-income, high-risk pregnant women. Informants shared that women migrated from various countries—many Caribbean and African countries—presenting in their third term of pregnancy and near delivery. One clinician explained:

Many walk in here straight off a plane. They’re far gone in the pregnancy and then umm with NO insurance.

There was emphasis on women appearing for services late in the pregnancy for the provision of care despite the lack of health insurance.

The provider also added:

We had a subset of patients who would travel here from out of the country, they would come here and a lot of them had their prenatal records, they would get emergency Medicaid, deliver, have their postpartum visit and then leave and go home

Social programs such as state Medicaid and PCAP make provisions for women who are pregnant to qualify for access to prenatal care. However, although such programs facilitate access to clinical prenatal care, we found that the women in our study more importantly emphasized the social determinants of prenatal health, including social systems and mHealth that provided support and information and improved participants’ prenatal health-seeking behaviors.

Women noted that the advice, information, and support from their circle of family, friends, and other pregnant women in Web-based chat groups made them feel more prepared for motherhood. For many women, the internet or other mHealth apps were a major source of prenatal health information. In the current age of mobile and digital technology, it is not surprising that interviewees unanimously mentioned extensive use of the internet, Google, and sites such as BabyCenter as primary go-to sources for prenatal health information and also to fact-check doctors. Participants were attracted to online forums and groups for pregnant women “with whom participants could relate” and communicate with to share and learn from others’ experience:

Sometimes you go in the chat rooms...you see people doing their methods of what works...but...it gives you something, it gives you a little more confidence too sometimes...you know...just to see the same amount of weeks or people going through the same symptoms that I am...

Similarly, a second participant expounded:

Yea there’s this app called baby prep baby pregnancy or something app, I have it on my phone...You talk to people all over the world...and all you have to do is put in your due date, they’ll like link you up with a bunch of people who are in your time in your pregnancy...and everybody have the same similarities...you know going through the same thing so you’ll feel more comfortable hearing from other people...around your time or whatever but doctor wise...I don’t know

There was a sense of trust, comfort, and pleasure with being able to go online for information, and many of the participants spoke of the increased access they have via their mobile phones. The women showed strongly positive attitudes toward the use of T4B and articulated that receiving push messages targeted specifically to their stages of pregnancy as a benefit that would even save them time from seeking information on their own.

### Quantitative Findings

#### Attitudes and Beliefs Statements

In general, initial attitudes toward T4B and key prenatal health behaviors were mostly neutral among pregnant women in the study, as indicated by a mean rank score of 3.71 on the attitude scale (alpha coefficient .661). A score of 4 would indicate overall agreement. Survey results show that approximately 10% of respondents neither agreed nor disagreed with the statements on the scale. Approximately 84% of the participants strongly agreed with the statement that eating 5 or more fruits and vegetables per day is important to the health of their baby, which reflect a 22% increase from pre- and post-test (*P*=.02). After T4B exposure, there was a 26% increase in the amount of women who strongly agreed that visiting their health care provider on a regular basis will help them be a healthy new mother (*P*=.03). There was also a 38% increase (*P*=.03) between presurvey and postsurvey in the proportion of participants who strongly agreed that using T4B will help them to have more support during pregnancy. During posttest, 51.0% of survey respondents strongly agreed with the statement “text4baby will help me to get new information about prenatal health” as opposed to 39% during pretest—reflecting an increase of nearly 27%. Although many participants neither agreed nor disagreed on whether relatives and those close to them would support the use of mHealth and T4B (20%), after exposure results showed a 12% increase in those who strongly agreed with that statement.

#### Perceived Usefulness and Perceived Behavioral Control Statements

The perceived usefulness of T4B improved in survey respondents after exposure to the text messages. Initially, a moderate proportion of participants neither agreed nor disagreed that the T4B messages will help to have a healthier pregnancy (26.5%). During the same time, 28.6% of respondents strongly agreed. However, at posttest, the proportion of participants who strongly agreed increased to 46.9% (*P*=.02). These results indicate a positive shift in attitude regarding T4B’s usefulness. In contrast, strong agreement with the statement “online sources are helpful for searching prenatal health information” declined from initial testing to follow-up (from 46.9% to 40.8%). At the same time, the proportion of respondents who neither agreed nor disagreed increased from 6.1% to 14.3%, indicating a shift to more neutral attitudes in the usefulness of T4B. The proportion of women who believed that they find it easy to receive prenatal health information on their mobile phone increased slightly from 57.1% to 59.2%. In addition, the proportion of women who strongly agreed that T4B messages will allow them to have greater control over their prenatal health care increased by 56% between pretest and posttest from 28.6% of participants to 51% (*P*=.02). However, in contrast, there was a slight increase in strong agreement that “I have the skills needed to use Text4baby,” and there was an increase from 4% to 14% in those having no opinion on that statement. Approximately 10% of the women surveyed agreed that reading English is sometimes difficult for them.

#### Compatibility, Relative Advantage, and Visibility Statements

A large percentage (85%) of respondents either agreed or strongly agreed with the compatibility of T4B messages by self-reporting regular use and communication via text messaging. A small portion (6%) either disagreed or strongly disagreed with the statement “I communicate regularly with friends and family through text messages,” suggesting high usage of text messaging for communication and a strong compatibility with T4B’s mode of disseminating information. Participant’s perceptions about the relative advantage of using T4B improved after receiving the T4B messages. Overall, participants agreed (mean score 4.15) with the items on the relative advantage scale. There were significant increases in the proportion of respondents who strongly agreed with the statement “using Text4baby will allow me to reach healthier prenatal health goals” and the proportion of respondents who initially had no opinion, indicated by them neither agreeing nor disagreeing with the statement decreased from 10% and 16% before using T4B to 6.1% post T4B. T4B had low visibility within our study participants. A small percentage (8.2%) reported having seen or heard of someone using T4B. A larger proportion of respondents neither agreed nor disagreed (30.6%) and others either disagreed or strongly disagreed (24.5% and 16.3%, respectively) about having seen or heard of T4B use.

#### Behavioral Intent to Use Text4baby

Study participants largely reported their intent to use the T4B program (rank score 4.28). A total of 47% and 46%, respectively, agreed and strongly agreed that they plan to use T4B for accessing prenatal health care and information.

Similarly, 91.8% of the participants strongly agreed to speak more to their doctor about information they learn through T4B.

## Discussion

### Mixed Findings and Implications (for Research, Policy, and Practice)

The number of mHealth educational interventions for pregnant women is rapidly evolving, but research in this area—although growing— is still limited. Before this study, there existed no knowledge as to what determinants influenced T4B usage intentions and if participants’ attitudes, beliefs, and perceptions would improve as a result of receiving the text messages. There are no studies that theoretically measure constructs of consumer health behavior, technology acceptance, and diffusion to conceptualize intent to use the T4B mHealth program. This is the first study to examine changes in attitudes, beliefs, and perceptions among urban African American and Afro-Caribbean immigrant pregnant women after exposure to T4B, and it provides novel insights by examining how T4B usage intentions may be influenced by perceived usefulness, relative advantage, perceived behavioral controls, and its compatibility within this study’s population.

Despite the growing number of research endeavors investigating mHealth and T4B [9,57,58], none have used a sequential exploratory mixed methods design incorporating qualitative phenomenology followed by repeated-measures pre-/post-test design around T4B intervention. Our investigation revealed that pregnant women often felt that the information they received during prenatal visits was not adequate at meeting their health communication needs; however, they believed that mHealth and T4B could increase their access to health care and information. When asked how receptive they were to using T4B and receiving prenatal health text messages on their cellphones, respondents replied:

I wouldn’t mind that cause...these phones now a days who don’t have messages just popping up out of everywhere; yea I think it great cause instead of like going to google...and trying to type you just receive a text and they tell you click the link I think it’s easier

Survey respondents were later asked to rate on a 5-point Likert scale their level of agreement with the statement “Text4baby will help me to get new information about prenatal health.” Although 51% of the participants strongly agreed, approximately 10% of the participants remained neutral after having received the T4B messages. A 2012 study of pregnant women attending public hospitals and antenatal care centers in Argentina found that a vast majority (95.9%) of the women reported willingness to receive SMS messages during pregnancy [59]. A study of pregnant women and health care professionals also revealed that pregnant women believed 3 SMS messages per week was an appropriate and preferred dose of SMS message to receive during pregnancy [9].

We found that pregnant women often placed greater value on their social support system over clinical prenatal care services for complete and quality care. This included family, peers, social networks, and online communities for pregnant women and government social programs such as the Woman, Infant, and Children nutritional assistance programs. This was most notable as many women expressed great dissatisfaction with the lack of engagement they have with providers. Other researchers have suggested that one potential explanation for improved outcomes amongst pregnant black women is the provision of social support, coping strategies, and stress reduction through group prenatal care [60].

With regard to care and information, responds alluded to using mHealth as a support to check information provided to them by doctors:

I even look up certain things that I don’t feel that’s right that the doctor, whatever the doctor say I look it over just to make sure they not giving the wrong information cause you know sometimes...people do make mistakes...you know...but...but just to make sure I’m ok and my baby’s is safe...I’ll look it over...do the research...that’s...that’s what it’s about the internet is everything for me lol.

Our findings extend prior research [61] which showed that quality prenatal care must equally weigh on other nonclinical factors, such as interpersonal care processes like attitude and emotional support; and structure of care including access and physical setting; and care provider characteristics as a part of quality clinical prenatal care. Overall, our findings corroborate with others to confirm high acceptability [16] and feasibility [17] for T4B and similar text messaging interventions for pregnant women. Given the high population of Afro-Caribbean immigrants with limited English proficiency and multiple dialects spoken, we believe that a tailored mHealth program should be considered for this population to supplement access to information and resources. Patient-centered approaches that leverage partnerships between health care providers and community-based organizations could provide patients with access to culturally competent doulas and other community health workers in a novel way to increase engagement, support, and educational opportunities during pregnancy.

### Future Implications

This research has a number of important implications for research, policy, and practice around mHealth and T4B. First, it provides a framework for more robust evaluation of the effects of T4B in this population of pregnant women by fostering an examination and prediction of T4B use through an initial assessment of patients’ knowledge and perceptions regarding its use. Second, the study of consumer health behavior and IT uses the factors associated with mHealth, and text message use provides strategic targets for prevention and intervention through the design of cogent strategies that encourage its use among patients at Downstate.

New York State Department of Health is currently in year 3 of a 5-year endeavor to redesign health care delivery systems for residents in the State Delivery Systems Reform Incentive Payment program. There is a renewed focus on nonclinical social determinants of health and the provision of value-based care by community health organizations that provide health education and promotion services for people with low socioeconomic status. This research implies opportunities for health policy decisionmakers to further investigate, develop, and implement nontraditional patient-centered prenatal health care services that are better positioned to address the many health, education, and communication barriers faced by low income pregnant women in Brooklyn New York. This research also implies the use of mHealth and text messaging to communication environmental health and prenatal risk assessment messages for women in Brooklyn; and for environmental and population health surveillance as early warning signs of emerging public health threats, and as emergency information systems in natural disasters or pandemics [36].

### Strengths and Limitations

The strengths of this study include the robust survey; sampling and analysis methods; and the triangulation of the qualitative data with focus groups, key informants, and observations. In addition, the development of a survey based on theoretically driven constructs of technology acceptance, innovation diffusion, and theory of planned behavior offers added strength. There are a number of limitations to this study, namely, the small sample size and the use of convenience sample, which can introduce sampling biases such as nonresponse and selection bias. This does not allow us to generalize to other populations of pregnant women; however, results may be indicative to similar urban and immigrant populations. The nature of pretest and posttest designs can also introduce biases due to response shift and maturation.

### Conclusions

T4B is a text messaging program that provides prenatal care messages to pregnant women and new mothers. It uses a partnership model with health care facilities often serving as local implementation partners [36]. Although mHealth interventions have been proposed as effective solutions to improve maternal and neonatal health [56], this study showed that the use of mHealth for prenatal health information was quite common, whereas internet searches, Google, and pregnancy-related app usage was most widespread. Receiving prenatal health electronic messages through texting is a positive avenue and highly compatible to provide pregnant women in central Brooklyn with information; however, more research with a larger population and direct modeling of testing of the theoretical constructs is needed to fully assess the perceived usefulness and relative advantage of T4B in this population. Although there was moderate intent to use the T4B program possibly because of its facilitation in women accessing information, gaining more control, and reaching healthier pregnancy goals, it is important that any mHealth endeavor must first be designed and tailored with the inclusion of those targeted to ensure that the messages and content are relevant and for a specific place-based population.

## Abbreviations

IT: information technology
LBW: low birth weight
mHealth: mobile health
RCT: randomized controlled trial
T4B: Text4baby

## References

[1] Li W, Huynh M, Lee E New York City Department of and Mental Hygiene.

[2] New York State Department of Health.

[3] Martin J, Hamilton BE, Osterman MJ, Curtin SC, Matthews TJ (2013). Births: final data for 2012. Natl Vital Stat Rep.

[4] Matthews TJ, MacDorman MF, Thoma ME (2015). Infant mortality statistics from the 2013 period linked birth/infant death data set. Natl Vital Stat Rep.

[5] Thomas SB, Fine MJ, Ibrahim SA (2004). Health disparities: the importance of culture and health communication. Am J Public Health.

[6] Freimuth VS, Quinn SC (2004). The contributions of health communication to eliminating health disparities. Am J Public Health.

[7] Attanasio L, Kozhimannil KB (2015). Patient-reported communication quality and perceived discrimination in maternity care. Med Care.

[8] Guendelman S, Broderick A, Mlo H, Gemmill A, Lindeman D (2017). Listening to communities: mixed-method study of the engagement of disadvantaged mothers and pregnant women with digital health technologies. J Med Internet Res.

[9] Huberty J, Dinkel D, Beets MW, Coleman J (2013). Describing the use of the internet for health, physical activity, and nutrition information in pregnant women. Matern Child Health J.

[10] Cené CW, Roter D, Carson KA, Miller ER, Cooper LA (2009). The effect of patient race and blood pressure control on patient-physician communication. J Gen Intern Med.

[11] Roter DL, Stewart M, Putnam SM, Lipkin M, Stiles W, Inui TS (1997). Communication patterns of primary care physicians. J Am Med Assoc.

[12] Rai A, Chen L, Pye J, Baird A (2013). Understanding determinants of consumer mobile health usage intentions, assimilation, and channel preferences. J Med Internet Res.

[13] Free C, Phillips G, Felix L, Galli L, Patel V, Edwards P (2010). The effectiveness of M-health technologies for improving health and health services: a systematic review protocol. BMC Res Notes.

[14] Free C, Whittaker R, Knight R, Abramsky T, Rodgers A, Roberts IG (2009). Txt2stop: a pilot randomised controlled trial of mobile phone-based smoking cessation support. Tob Control.

[15] Fukuoka Y, Vittinghoff E, Jong SS, Haskell W (2010). Innovation to motivation--pilot study of a mobile phone intervention to increase physical activity among sedentary women. Prev Med.

[16] Aranda-Jan CB, Mohutsiwa-Dibe N, Loukanova S (2014). Systematic review on what works, what does not work and why of implementation of mobile health (mHealth) projects in Africa. BMC Public Health.

[17] (2014). US Department of Health and Human Services.

[18] Gazmararian JA, Yang B, Elon L, Graham M, Parker R (2012). Successful enrollment in Text4Baby more likely with higher health literacy. J Health Commun.

[19] Ferrer-Roca O, Cárdenas A, Diaz-Cardama A, Pulido P (2004). Mobile phone text messaging in the management of diabetes. J Telemed Telecare.

[20] Wallwiener S, Müller M, Doster A, Laserer W, Reck C, Pauluschke-Fröhlich J, Brucker SY, Wallwiener CW, Wallwiener M (2016). Pregnancy eHealth and mHealth: user proportions and characteristics of pregnant women using Web-based information sources-a cross-sectional study. Arch Gynecol Obstet.

[21] Jang J, Dworkin J, Hessel H (2015). Mothers' use of information and communication technologies for information seeking. Cyberpsychol Behav Soc Netw.

[22] Dobson R, Whittaker R, Bartley H, Connor A, Chen R, Ross M, McCool J (2017). Development of a culturally tailored text message maternal health program: TextMATCH. JMIR Mhealth Uhealth.

[23] Abroms LC, Johnson PR, Heminger CL, Van Alstyne JM, Leavitt LE, Schindler-Ruwisch JM, Bushar JA (2015). Quit4baby: results from a pilot test of a mobile smoking cessation program for pregnant women. JMIR Mhealth Uhealth.

[24] Bahanshal S, Coughlin S, Liu B (2017). For You and Your Baby (4YYB): adapting the centers for disease control and prevention's Text4Baby program for Saudi Arabia. JMIR Res Protoc.

[25] Bushar JA, Kendrick JS, Ding H, Black CL, Greby SM (2017). Text4baby influenza messaging and influenza vaccination among pregnant women. Am J Prev Med.

[26] Evans WD, Wallace JL, Snider J (2012). Pilot evaluation of the text4baby mobile health program. BMC Public Health.

[27] Huberty J, Rowedder L, Hekler E, Adams M, Hanigan E, McClain D, Balluff M, Buman M, Bushar J (2016). Development and design of an intervention to improve physical activity in pregnant women using Text4baby. Transl Behav Med.

[28] Remick AP, Kendrick JS (2013). Breaking new ground: the text4baby program. Am J Health Promot.

[29] van Velthoven MH, Majeed A, Car J (2012). Text4baby - national scale up of an mHealth programme. Who benefits?. J R Soc Med.

[30] Ybarra ML, Holtrop JS, Bağci Bosi AT, Emri S (2012). Design considerations in developing a text messaging program aimed at smoking cessation. J Med Internet Res.

[31] Lichtenstein E, Zhu S, Tedeschi GJ (2010). Smoking cessation quitlines: an underrecognized intervention success story. Am Psychol.

[32] Johnson WD, Diaz RM, Flanders WD, Goodman M, Hill AN, Holtgrave D, Malow R, McClellan WM (2008). Behavioral interventions to reduce risk for sexual transmission of HIV among men who have sex with men. Cochrane Database Syst Rev.

[33] Gaglio B, Smith TL, Estabrooks PA, Ritzwoller DP, Ferro EF, Glasgow RE (2010). Using theory and technology to design a practical and generalizable smoking reduction intervention. Health Promot Pract.

[34] Almatari A, Noorminshah A, Ali S (2013). Journal of Information Systems Research and Innovation (JISRII)5 (2013).

[35] Marton C, Wei Choo C (2012). A review of theoretical models of health information seeking on the web. J Doc.

[36] Sondaal SF, Browne JL, Amoakoh-Coleman M, Borgstein A, Miltenburg AS, Verwijs M, Klipstein-Grobusch K (2016). Assessing the effect of mHealth interventions in improving maternal and neonatal care in low-and middle-income countries: a systematic review. PLoS One.

[37] Warren J, Kvasny L, Hecht M, Burgess D, Ahluwalia J, Okuyemi K (2009). Barriers, control and identity in health information seeking among African American women. J Health Dispar Res Pract.

[38] Ajzen I (1985). From Intention to Actions: A theory of Planned Behavior. Action Control.

[39] Ajzen I (1991). The theory of planned behavior. Organizational Behavior and Human Decision Processes.

[40] Davis FD (1989). Perceived usefulness, perceived ease of use, and user acceptance of information technology. MIS Q.

[41] Davis FD (1993). User acceptance of information technology: system characteristics, user perceptions and behavioral impacts. Int J Man Mach Stud.

[42] Rogers E (2003). Diffusion of innovations5.1.38 (2003).

[43] Geer LA, Persad MD, Palmer CD, Steuerwald AJ, Dalloul M, Abulafia O, Parsons PJ (2012). Assessment of prenatal mercury exposure in a predominately Caribbean immigrant community in Brooklyn, NY. J Environ Monit.

[44] Thomson D, Bzdel L, Golden-Biddle K, Carole A, Reay, Trish & Estabrook (2005). Central questions of anonymization: a case study of secondary use of qualitative data. Forum Qual Soc Res.

[45] Dill LJ (2017). "Wearing My Spiritual Jacket": the role of spirituality as a coping mechanism among African American youth. Health Educ Behav.

[46] Hubbard W, Sandmann L (2007). Using diffusion of innovation concepts for improved program evaluation. Journal of Extension.

[47] Yu CH (2008). Designing and Conducting Mixed Methods Research. Organizational Research Methods.

[48] Leard Dissertation.

[49] Creswell JW, Hanson WE, Clark Plano VL, Morales A (2016). Qualitative research designs. Counsel Psychol.

[50] Fitzgerald EM, Cronin SN, Boccella SH (2016). Anguish, yearning, and identity: toward a better understanding of the pregnant Hispanic woman's prenatal care experience. J Transcult Nurs.

[51] Merriam S, Tisdell EJ (2015). Qualitative Research: A Guide to Design and Implementation, 4th Edition.

[52] Phan K, Daim T (2011). Exploring technology acceptance for mobile services. J Ind End Manag.

[53] Evans W, Nielsen PE, Szekely DR, Bihm JW, Murray EA, Snider J, Abroms LC (2015). Dose-response effects of the text4baby mobile health program: randomized controlled trial. JMIR Mhealth Uhealth.

[54] Colaizzi PF (1978). Psychological research as the phenomenologist views. Existential-Phenomenological Alternatives for Psychology.

[55] Miles M, Huberman M, Saldana J (2013). Sage publications.

[56] Geer LA, Curbow BA, Anna DH, Lees PS, Buckley TJ (2006). Development of a questionnaire to assess worker knowledge, attitudes and perceptions underlying dermal exposure. Scand J Work Environ Health.

[57] Foster J, Miller L, Isbell S, Shields T, Worthy N, Dunlop AL (2015). mHealth to promote pregnancy and interconception health among African-American women at risk for adverse birth outcomes: a pilot study. Mhealth.

[58] Cormick G, Kim NA, Rodgers A, Gibbons L, Buekens PM, Belizán JM, Althabe F (2012). Interest of pregnant women in the use of SMS (short message service) text messages for the improvement of perinatal and postnatal care. Reprod Health.

[59] Sword W, Heaman MI, Brooks S, Tough S, Janssen PA, Young D, Kingston D, Helewa ME, Akhtar-Danesh N, Hutton E (2012). Women's and care providers' perspectives of quality prenatal care: a qualitative descriptive study. BMC Pregnancy Childbirth.

[60] Scott KA, Britton L, McLemore MR (2019). The ethics of perinatal care for black women: dismantling the structural racism in "Mother Blame" narratives. J Perinat Neonatal Nurs.

[61] Whittaker R (2012). Issues in mHealth: findings from key informant interviews. J Med Internet Res.

